# Impact of health education intervention on insecticide treated nets uptake among nursing mothers in rural communities in Nigeria

**DOI:** 10.1186/1756-0500-5-444

**Published:** 2012-08-18

**Authors:** Olorunfemi E Amoran, Kehinde O Fatugase, Olubunmi M Fatugase, Kabir O Alausa

**Affiliations:** 1 Department of Community Medicine and Primary Care, College of Health Sciences, Olabisi Onabanjo University Teaching Hospital, Sagamu, Nigeria

**Keywords:** ITN, Utilization, Malaria, Health education intervention, Nursing mothers, Rural Nigeria

## Abstract

**Background:**

ITN use is generally poor in Nigeria among all categories of people. Although use of ITNs has been shown to reduce malarial morbidity and mortality, this measure needs to be supported by an adequate healthcare system providing ITN possibly at the household level. This study was therefore designed to determine the effect of health education on the uptake of ITN among nursing mothers in rural communities in Nigeria.

**Methods:**

The study design was a quasi-experimental study carried out in Ijebu North Local Government Area of Ogun State. A multistage random sampling technique was used in choosing the required samples for this study and a semi- structured questionnaire was used to collect relevant information. The intervention consisted of a structured educational programme based on a course content adapted from the national malaria control programme. A total of 400 respondents were recruited into the study with 200 each in both the experimental and control groups and were followed up for a period of 3 months when the knowledge and uptake of ITN was reassessed.

**Result:**

There was no significant difference (P >0.05) observed between the experimental and control groups in terms of socio-dermographic characteristics such as age, marital status, religion, and income. The ITN ever users in experimental group were 59 [29.5%] and 138 [72.6%] in pre and post intervention period, respectively (p value =0.0001). These proportions of ITN ever users were 55 [27.5%] and 57 [31.6%] in control group, during the pre and post intervention periods (p = 0.37). Post health education intervention, degree of change in knowledge of ITN re-treatment [37.0%] and mounting [33.5%], readiness to use if given free [30.5%] and belief in efficacy [36.9%] improved significantly in the experimental group while there was no significant change in the control group [p = 0.84, 0.51, 0.68 &0.69 respectively]. Majority [89%] of the respondents were willing to buy ITN for between US$ 1.5 to US$ 3.0. There was no statistically significant change (P >0.05) despite intervention in the amount the respondents were willing to pay to own an ITN in both the experimental and control groups.

**Conclusion:**

The study concludes that the use of ITN in the study population was significantly increase by health education and that the free distribution of ITN may not guarantee its use. Uptake of ITN can be significantly improved in rural areas if the nets are made available and backed up with appropriate health education intervention.

## Background

Malaria constitutes a major economic burden in the sub-Sahara Africa including Nigeria [1,2]. In some countries with a heavy malaria burden, the disease may account for as much as 40% of public health expenditure, 30-50% of inpatient admissions, and up to 50% of outpatient visits [3,4]. Malaria has significant measurable direct and indirect costs, and has recently been shown to be a major constraint to economic development [5-9]. The ultimate aim of Behavioural Change Communication (BBC) is to ensure that individuals, families, communities and health workers are taking preventive measures against malaria especially regular use of ITN [10]. A control strategy comprising of proper application of existing means is advocated. This include Early Diagnosis and Treatment (EDT) of symptomatic malaria to prevent progression to severe and potentially fatal stages, preventive measures including use of ITNs and selective residual spraying and prediction, containment and if possible, prevention of epidemics and strengthening of local capacities [11]. WHO advocates the combined approach of ITNs and EDT in its Roll Back Malaria initiative [11] ITN utilization is generally poor in Nigeria among all categories of people [1,6,7]. It was observed that only 12% of households in Nigeria reported owning mosquito nets [3,12]. A study conducted in Nsukka, Enugu State revealed that 22% of the survey respondents reported that bed nets had been used before by a member of their household [2]. Willingness to pay for bed nets and insecticides for treating them was found to be high in four communities in Eastern Nigeria [13,14]. The recently published Nigeria Demographic and Health Survey (NDHS 2003) revealed that 12% of all households own at least a net (any type) while only 2% own at least one ITN [1,8,9]. There is also a wide variation of net ownership across the six geo-political zones with more nets being available among the households in the North (except North East) than the South. Trials of insecticide-treated mosquito nets (ITNs) in the 1980s and 90s showed that ITNs reduced deaths in young children by an average of 20% [15-17]. One of the targets set at the Abuja Summit in April, 2000 was to have 60% of populations at risk sleeping under ITNs by 2005 [1,8,18]. This will require 32 million mosquito nets and a similar number of insecticide re-treatments each year [18]. To achieve this, much work still needs to be done to make ITNs affordable, widely available, and most importantly, improving utilization among the consumers. This study was therefore designed to determine the effect of health education on the uptake of ITN among nursing mothers in rural communities in Nigeria.

## Methodology

### The study area

The study was carried out in Ijebu North Local Government Area of Ogun State. Ijebu North Local Government is one of the twenty Local Government in Ogun State. The experimental study was carried out in Oru a semi-rural town in Ijebu North Local Government Area, of Ogun State Nigeria. It is bounded in the East by Iperin, West by Awa, North by Ijebu-Igbo, and South by Ago Iwoye. Oru has a population of about 100,000 people (2006 population census). The control study was carried out in Atikori ward at Ijebu-Igbo, a semi-rural town in Ijebu North Local Government Area, of Ogun State Nigeria with a population of about 150,000 people (2006 population census) [19].

### Study design

The study design was a quasi-experimental study to determine the effect of malaria education programme on the uptake of ITN among nursing mothers in rural communities in Nigeria. Two political wards, one randomly selected from the southern axis (Ijebu-Oru) and the other one randomly selected from the northern axis (Ijebu-Igbo) formed the experimental and control groups respectively. It was decided to choose the experimental and control groups from two different ends (North axis and South axis) of the Local Government to prevent cross interference during and after the intervention periods. The distance between the experimental and the control group is about 10 km.

The study was carried out in three phases – Pre-intervention, Intervention and Post-Intervention phases. Phase one (pre- intervention) involved cross-sectional comparative descriptive study, while phase two involved comprehensive health education intervention in the experimental group only. Phase three (post-intervention) involved comparative study between the experimental and control group.

### Sample size

The minimum sample size needed was obtained from the formula for comparing proportions between two groups^40^.

$$n={\left\{(,Z_{-\alpha /2},\sqrt{2PO\left(1-PO\right)},\;,Z_{\beta },\;,\sqrt{PO\left(1-PO\right)+P1\left(1-p_{1}\right)},),\;,P,O,-,P_{1}\right\}}^{2}$$

The outcome measure for computing the sample size was the proportion of households in Nigeria using mosquito nets, P1 = 12% (NDHS, 2003).

The study was hoped at improving the percentage by 15%

P2 = Minimum proportion of mothers expected to be utilizing mosquito net after the intervention = 27%

P0 = average of p1 and p2 = (12 + 27)/2 = 19.5%

Z_1- α/2_ = Standard normal deviate corresponding to level of significant (α) of 5% = 1.96

Z_β_ = Standard normal deviate corresponding to type II error of 10% (Power = 90%) = 1.28.

D = design effect of 1.5 for the sampling design used

P1-P2 = 15%

Then

$$n=1.5{\left\{\begin{array}{l} 1.96\sqrt{2\times 0.195\left(1-0.195\right)+1.28}\sqrt{0.12\left(1-0.12+0.27\right)\left(1-0.27\right)}\\ \;0.15\; \end{array}\right\}}^{2}$$

The minimum sample size from the above formula is 182 for each group. However 200 women per group were studied after allowing for 10% attrition rate.

### Subject selection

(i) **Inclusion Criteria:** Only mothers or guardians who are permanent residents (resident in the area >6 months) and currently having children of <5 yrs of age living with them were included in the study.

(ii) **Exclusion Criteria:** Mothers or guardians whose <5 yrs old children were not living with them at the time of the study were not included in the study.

### Sampling technique

A multistage random sampling technique was used in choosing the required samples for this study. Ijebu North Local Government has seven political wards. Four of these wards were located in the Northern axis of the local government and the remaining three were in the Southern axis of the Local Government. Each of the political wards served as a cluster. The first step was to choose between the northern part and the southern part which one became the experimental or control group, this was done by tossing a coin. From the list of political wards in each axis, a ward was selected by simple random sampling technique by casting a lot e.g. balloting using same size of papers and thoroughly mixed and then picking it at random. House enumeration was carried out by the researcher and two officials from the town-planning unit of the Local Government. A total number of one thousand, and eight hundred houses were counted in the experimental and control wards respectively. A systematic random sampling technique using a sample interval of five and four in the experimental and control wards respectively was used to choose two hundred houses each in experimental and control groups respectively. The sample interval was obtained by dividing the total number of houses by the sample size in the experimental and control wards respectively (1000/200 and 800/200}. The first house was determined by using the table of random number to pick a house from the house enumeration list and the one household was studied per house and this was randomly selected. In the two groups, a simple random sampling technique was carried out by ballotment to choose a mother of under five from an household where there were more than one mother with under five in a house. Where there was one household in a house, the mother of under five automatically qualified to participate in the study.

### Data collection

#### Pre-intervention activities

The local LGA authorities were approached to obtain official permission to proceed with the project. Mothers of under-five children who consented to take part in the study were interviewed using a structured questionnaire which was administered by trained interviewers. Fifty households were selected in a nearby community (Ilaporu) for pre-testing of the questionnaire before large scale study. The questionnaires were pre-tested with the research assistants, who had debriefing on field experiences and proffered solutions to identified problems. Amendments were made, which led to re-designing aspects of the instrument that were ambiguous or lacked clarity. A baseline survey to determine the mothers knowledge, attitude and practice (KAP) about malaria prevention and management was conducted using the corrected questionnaires. This represented the pre-training assessment for the intervention group and the initial assessment for the control group. An average of 20 questionnaires were administered daily for 10 days. The same was also done for the control group.

#### Intervention activities

The intervention consisted of a structured educational programme based on a course content adapted from the national malaria control programme and the information obtained from the gaps in knowledge identified from the distributed questionnaire formed the basis of the training. Training sessions were conducted during which various aspects of the management and control of malaria were taught. Multiple health channels were used. These include: A training workshop, Use of education materials such as posters, story book, and malaria post signs. Two malaria post signs were erected at the community health centre, which is beside the community major market. The signs post indicated graphic descriptions of the insecticide treated bed net and directions for its use. The benefits and annotations was written in Yoruba. The sign post was located at conspicuous positions around the health centre, which is not far from the major market. Colourful malaria posters indicating malaria symptoms and signs in children and annotated diagrams for prevention and treatment was pasted at different locations within the health centre.

Each batch was trained for one day. The training consisted of 3 modular units which are; knowledge about malaria transmission, its prevention and treatment; attitude on malaria prevention strategies; practice of malaria prevention and treatment practices. Each module consisted of a lecture and an exercise. The training period lasted for two weeks with training taking place five days a week. The participating mothers/guardians were divided into 10 batches of 20.

### Post-intervention

The post-intervention evaluation was carried out to determine a residual gain in malaria-related KAP three months after the training and initial assessment in the intervention and control groups respectively. This represented the three months post-training assessment. Evaluation of the effects of training was done using standardized scores for the various variables during analysis.

### Data analysis

The questionnaires were kept safe and confidential and checked for proper completion on collection from participants. The data was entered into SPSS statistical software version 12. Frequencies were generated for detection of errors (data editing). Data was summarized using means, standard deviation and proportions.

To measure the effectiveness of health education intervention, the degree of change was measured and this was subjected to the tests of significance (McNemar’s Chi-square, P-values) where appropriate. The degree of change between two samples was calculated by finding the difference in percentage point between the proportions in the second sample with a given attribute and the proportion in the first sample with the same attribute. This was calculated in both the experimental and control groups.

For the purpose of analysis, marital status was re-categorized as currently married and not married. Not married include single, the separated and the widows. Knowledge of malaria was categorized as good and poor: “good response” entailed the knowledge that malaria is caused by mosquito insect while other responses regarding malaria causation were categorised as “poor” level of knowledge.

### Ethical consideration

The research proposal was approved by the Olabisi Onabanjo University Teaching Hospital Ethical Committee. Informed consent was obtained from the Chairman, Ijebu North Local Government Area and the community leaders. Oral and written consent was obtained from the selected mothers and guardians before administering the questionnaires.

## Results

### Socio-dermographic characteristics of respondents

The control group had two hundred respondents (50% of the total number of participants); 180 (90%) of them were available to complete the questionnaire after the three-month intervention period. The experimental group had two hundred respondents (50% of the total number of participants) of which 190 (95%) responded to the study questionnaires after the three-month intervention period. The socio-demographic characteristics of the caregiver in both the experimental and control groups are shown in Table 1.

**Table 1:** Socio-Demographic Characteristics of the Respondents

	**Experimental group N = 200 (%)**	**Control group N =200 (%)**	**Test statistic value (X**^**2**^**)**	**p – value**
**Age in years**			0.02	0.99
<25	52 (26.0)	53 (26.5)		
25 – 34	105(52.5)	105 (52.5)		
35+	43(21.5)	42(21.0)		
**Total**	**200 (100)**	**200 (100)**		
**Marital Status**				
Currently married	184 (92.0)	180(90.0)	0.49	0.48
Others	16(8.0)	20 (10.0)		
**Total**	**200 (100)**	**200 (100)**		
**Religion**				
Christianity	133 (66.6)	148 (74.0)	2.68	0.1
Islam	67(33.3)	52 (26.0)		
**Total**	**200 (100)**	**200 (100)**		
**Mother’s income (Naira)**				
Less than 2500	66 (33.0)	74 (37.0)	2.33	0.51
2500-4999	64 (32.0)	59 (29.5)		
5000-7499	27 (18.5)	19 (9.5)		
7500+	43 (21.5)	48 (24.0)		
**Total**	**200 (100)**	200 (100)		
**Father’s income (Naira)**				
Less than 2500	11(5.5)	12(6.0)	1.13	0.77
2500-4999	19(9.5)	23(11.5)		
5000-7499	38(19.0)	43(21.5)		
7500+	132(66.0)	122(61.0)		
**Total**	200 (100)	**200 (100)**		

There was no significant statistical differences observed between the experimental and control groups in terms of sociodermographic characteristics such as age [p = 0.99], marital status [p = 0.48], religion [p = 0.1], and income [p = 0.51].

### Participants’ utilization of Insecticide Treated Nets (ITNs)

The ITN ever users in experimental group were 59 [29.5%] and 138 [72.6%] in pre and post intervention period, respectively (p value =0.0001). These proportions of ITN ever users were 55 [27.5%] and 57 [31.6%] in control group, during the pre and post intervention periods (p = 0.37). Knowledge of disease causation improved in the experimental group from 69.4% to 95% [p <0.001] but remained significantly stable from 73.3% to 72.3% [p = 0.78] among the control group. Table 2 showed that in the experimental group, 129 [64.5%] of the respondents were knowledgeable about the process of mounting the ITNs and after the intervention, the level of knowledge significantly increased to 186 [97.9%] of the respondents, this showed 33.5% points degree of change (p <0.001). However, the knowledge of participants about the mounting of ITN only improved slightly in the control from 45.5% to 49.0% [p = 0.51].

**Table 2:** Respondents’ Utilization of Insecticide Treated Nets (ITNs)

	**Experimental group**				**Control group**			
	**Pre intervention N = 200 [%]**	**Post intervention N = 190 [%]**	**Degree of change**	**P value**	**Pre intervention N = 200 [%]**	**Post intervention N = 180 [%]**	**Degree of change**	**P value**
**Prevalence of ITN use**								
Ever use ITN	59 [29.5]	138 [72.6]				45.2	<0.001	55 [27.5]	57 [31.6]	4.1	0.37
Never use ITN	141 [70.5]	52 [27.4]						145 [72.5]	123 [68.4]		
**Amount willing to pay to own ITN**											
<N200	82 [41.0%]	84 [44.2]				3.2	0.47	88 [44.0%]	82 [45.6]	−1.6	0.93
N200-N500	84 [42.0%]	82 [43.2]						90 [45.0%]	80 [44.4]		
N500-N1000	34 [17.0%]	24 [12.6]						22 [11.0%]	18 [10.0]		
**Knowledge about mounting ITN**											
Knowledgeable	129 [64.5]	186 [97.9]				33.5%	<0.001	91 [45.5]	88 [48.9]	−3.4%	0.51
Not Knowledgeable	69 [35.5]	4 [2.1]						109 [54.5]	92 [51.1]		
**Knowledge of treatment of ITN**											
Correct (6 monthly )	78 [39.0]	144 [75.8]				36.8	<0.001	34 [17.0]	32 [17.8]	0.8	0.84
Incorrect	122 [61.0]	46 [24.2]						166 [83.0]	148 [82.2]		

Only 78 [39.0%] in the experimental group knew when ITN should be re-treated before the intervention. After the intervention, the level rose to 144 [76.0%] of the respondents which showed 37.0% points degree of change (p <0.001). On the other hand in the control group the value remain almost the same in the pre 34(17.0%) and post −32(17.8%) intervention period [p = 0.84].

Table 2 shows that about 166 [83%] and 178 [89%] of mothers in the experimental and control group respectively were willing to own an ITN if it is sold between <200 and 500 [$1.5 and $3]. Amount of money mothers were willing to pay for ITN in both the experimental and control group remain significantly constant in both pre and post intervention phase of the study [p = 0.47 & 0.93 resp.].

### Attitude to ITN utilization and malaria prevention

Tables 3 showed that pre-intervention 137 [69.4%] of the respondents had correct knowledge of aetiology of malaria with a significant increase to 100% [p <0.001] post intervention in experimental group. Among the controls, there was no difference in the pre and post intervention proportions of the respondents knowledge of malaria aetiology [p = 0.78].

**Table 3:** Respondents’ attitude to ITN and Malaria prevention

**Experimental group**					**Control group**			
	**Pre intervention N = 200 [%]**	**Post intervention N = 190 [%]**	**Degree of change**	**P value**	**Pre intervention N = 200 [%]**	**Post intervention N = 180 [%]**	**Degree of change**	**P value**
**Knowledge of disease causation**								
Correct	137 [68.5]	180 [95.0]	26.5	<0.001	147 [73.5]	130 [72.2]	−1.3	0.78
Incorrect	63 [31.5]	10 [5.0]			53 [26.5]	50 [27.8]		
**Belief in the Efficacy of ITN**								
Efficient	121 [60.5]	185 [97.4]	36.9	<0.001	124 [62.0]	108 [60.0]	2.0%	**0.69**
Not efficient	**79 [39.5]**	**5 [2.6]**			**76 [38.0]**	**72 [40.0]**		
**Readiness to use ITN if given free**								
Yes	139 [69.5]	190 [100]	30.5		144 [72.0]	133 [73.9]		
No	61 [30.5]	0 [0.0]		<0.001	56 [28.0]	47 [26.1]	1.9	0.68

The attitudes to malaria prevention are shown in Table 3. The proportion of those willing to use ITN if given free improved significantly from 137 [69.5%] to 190 [100%] [p <0.001] in the pre and post intervention phase of the experimental group while there was no significant difference in the pre and post intervention phase in the control group (p = 0.68). There was an increase with health educational intervention in the belief in the efficacy of ITN [from 60.5% to 97.4%] in the experimental group [P <0.0001] and with very little changes in the control group over the 3 months where there was no health education intervention [p = 0.69].

## Discussion

This study shows a statistically significant increase in ITN utilization after health education in the experimental group. Utilization however remains almost the same despite the free distribution of ITN in the control group during the pre and post intervention phases. Factors that may influence the use of ITN has been shown to be related to the cost, and availability [20,21]. However, this study has shown that free distribution of ITN may not guarantee its use. It further emphasise the essential role of health education campaign in the fight against eradication of malaria in Africa. The prevalence of ITN ever use in the study was 29.8% and 27.8% in the experimental and control group respectively in the pre intervention phase The results of this study are comparable to several surveys in Africa, where ITN use varies from 5% to 70% depending on the population studied [22-27].

This study strongly demonstrated the effect of health education on improved correct knowledge and attitude about Malaria. After the intervention, the proportion of respondents in the experimental group who had the correct knowledge of aetiology of malaria increased significantly to 100%. This finding agreed with the findings of several other studies [25,28]. A health education interventional study of this nature is not only a veritable tool in primary control of an endemic communicable disease such as malaria, it also relates to all aspects of health behaviour including the use of health services. This study has proven the effect of this on mothers' behaviour in a rural setting.

The proportion of those who believed in the efficacy of ITN in preventing malaria improved significantly in the post intervention phase of the experimental group (p <0.001) while there was no significant difference in the post intervention assessment of the control group. Several studies have emphasised the efficacy of ITN [29-32], this have to be communicated to caregivers in the communities especially in rural areas if this fact will be properly utilized. Although use of ITNs has been shown to reduce malarial morbidity and mortality, this measure needs to be supported by an adequate healthcare system providing health education, possibly at the household level [33].

The fact that those ready to use ITN if given free improved significantly in the post intervention of the experimental group shows that cost is not the only barrier to their effective use [30-32]. Often people who are unfamiliar with ITNs, or who are not in the habit of using them, need to be convinced of their usefulness and persuaded to re-treat the nets with insecticide on a regular basis. WHO has worked with mosquito net and insecticide manufacturers to make re-treatment as simple as possible [31]. The fact that there was an 100% improvement in the number of participants in the experimental group that knew when and how the net should be re-treated in this study indicate that health education can help in this regard. However, the best hope lies with newly developed, long-lasting treated mosquito nets which may retain their insecticidal properties for four to five years (the life span of the net), thus making re-treatment unnecessary.

Majority [83% and 89%] of mothers in the experimental and control group respectively were willing to own an ITN if it is sold between <200 and 500 [between US$1.5 and $3]. Presently ITN is being distributed freely all over the state to every household in Ogun state at the time of this study by the National Malaria Control Programme. For sustenance of ITN use the price of ITN is essential. This study shows that the present price [US$3.5] of ITN in Nigeria is expensive for families at risk of malaria, who are among the poorest in the world. Similar studies [14,34] have observed similar findings in rural areas in Africa which noted that about 30% of their respondents were willing to pay for bed nets and actually redeemed their pledges [14]. With these economic restrictions, sustenance of this free distribution may be crucial to the control of Malaria disease in this region. Donor assistance, on a much larger scale than has been provided in recent years, may be necessary.

Given the nature of the experimental study, interpretation of study results should be done with caution. The study might also have been faced with a lot of influence from external forces such as the unforeseen introduction of the free distribution of the ITN by the state Ministry of Health after the conduct of the intervention phase in both communities which was a desirable effect. Other uncontrollable intervention might have occurred which might have introduced bias into the study. The prevention of the cross-over effect could not be totally guaranteed between the experimental and control groups during and after the intervention programme.

## Conclusion

The study concludes that the use of ITN in the study population was significant increase by health education and that the free distribution of ITN may not guarantee its use. It also demonstrated the effect of health education on improved correct knowledge and attitude about ITN. Uptake of ITN can be significantly improved in rural areas if the nets are made available and backed up with appropriate health education intervention.

## Competing interest

The authors declare that they have no competing interests.

## Authors’ contributions

FOK conceived the study and participated in its design, AOE participated in the analysis and helped to draft the manuscript, AOK & FBO participated in the coordination. All authors read and approved the final manuscript.
